# Feasibility of home-based transcranial direct current stimulation combined with personalized word retrieval for improving naming in primary progressive aphasia

**DOI:** 10.3389/fneur.2025.1543712

**Published:** 2025-02-11

**Authors:** Allan George, Eric McConathey, Amy Vogel-Eyny, Elizabeth Galletta, Giuseppina Pilloni, Leigh Charvet

**Affiliations:** ^1^Department of Neurology, New York University Grossman School of Medicine, New York, NY, United States; ^2^Rusk Rehabilitation, New York University Langone Health, New York, NY, United States

**Keywords:** aphasia, tDCS, telemedicine, home-based tDCS, SLT, primary progressive aphasia, naming

## Abstract

**Background/Objectives:**

Primary progressive aphasia (PPA) is managed with speech-language therapy (SLT) to slow language decline. Pairing transcranial direct current stimulation (tDCS) with SLT can enhance its effects. However, further research is needed to confirm these findings and guide its clinical use. We evaluated the feasibility of providing an intervention combining tDCS with SLT as a home-based and remotely supervised intervention.

**Methods:**

Participants with confirmed PPA who had word-finding difficulties were recruited for an open-label observational study. The intervention consisted of 20 daily sessions over 1 month, each with 45-min of personalized word retrieval training. During the first 30-min, participants received tDCS over the left inferior frontal gyrus (anode F7, cathode O1) at 2.0 mA. Language measures were remotely administered at baseline and intervention end.

**Results:**

We enrolled 10 patients (age: 70 ± 7 years; 60% male) with confirmed logopenic variant (*n* = 2), semantic variant (*n* = 2), or unspecified (*n* = 6) PPA. The intervention was well-tolerated with no treatment-limiting adverse events. All participants completed all sessions, confirming the feasibility of the home-based treatment. There were no declines in language functioning measures, with improved naming for trained vs. untrained items (*p* = 0.003) and a significant improvement in confrontation naming (*p* = 0.016) from baseline to intervention end.

**Conclusions:**

Our case series demonstrates that home-based tDCS added to SLT is feasible for patients with PPA. However, larger controlled studies are required to confirm its effectiveness in slowing language decline and to fully determine the benefits of this approach. This approach not only facilitates broader access to participation but also enables the extended treatment necessary to evaluate its clinical benefits, moving this treatment closer to clinical availability as a telehealth treatment.

## 1 Introduction

Primary progressive aphasia (PPA) is a neurocognitive disorder characterized by the gradual and progressive loss of language abilities, including speaking, understanding, reading, and writing, as a result of neurodegeneration (1). It is considered a neurodegenerative syndrome where language impairment is the most prominent and early symptom, differentiating it from other cognitive disorders (1). There are three main subtypes of primary progressive aphasia (PPA), each characterized by distinct language impairments and neuroanatomical patterns of atrophy (2). Semantic variant PPA (svPPA) involves a gradual loss of word and object meanings, resulting in fluent yet vague or empty speech, and is associated with atrophy in the anterior temporal lobes, predominantly on the left (3). Logopenic variant PPA (lvPPA) is marked by impaired word retrieval, speech hesitations, and difficulty repeating complex sentences, with atrophy localized to the left angular gyrus, the posterior third of the left middle temporal gyrus, and the left superior temporal sulcus (4). Nonfluent/agrammatic variant PPA (nfvPPA) manifests as effortful speech, grammatical errors, and difficulty constructing sentences, typically linked to atrophy in the left posterior frontal lobe, including the inferior frontal gyrus (IFG) and insula (5, 6).

These subtypes are associated with specific patterns of brain atrophy, which aid in diagnosis and management (2). Currently, there is no cure for PPA, but it is managed primarily through speech-language therapy (SLT), which aims to preserve language functions and slow disease progression (7). SLT for PPA typically involves personalized interventions such as lexical retrieval training, script training, and functional communication strategies. These approaches target specific linguistic challenges and employ techniques like semantic, orthographic, and phonological cueing to optimize preserved abilities and support compensatory communication. Evidence underscores the importance of tailoring SLT to the unique profiles of language impairment in PPA, including its logopenic, semantic, and nonfluent variants (7, 8). Group-based interventions and creative non-verbal strategies, as highlighted by Watanabe et al. (9), also contribute to psychosocial wellbeing and support linguistic processes in PPA.

While these therapies yield moderate benefits, they are typically limited to the trained domains, and progressive language decline remains inevitable over time. This highlights the need for complementary strategies, such as neuromodulation therapies like transcranial direct current stimulation (tDCS), which have shown promise in enhancing SLT outcomes by promoting neural plasticity and stabilizing language functions (10, 11).

The presumed mechanism of action of tDCS is the modulation of the ongoing neuronal activity of the region where current is directed (12). Although the induced electric field is not strong enough to trigger an action potential, it can still alter neuronal polarization, increasing synaptic efficacy and promoting long-term potentiation (LTP) (13). When applied for functional recovery, the current is directed to the region engaged in a training activity (“brain state”) (14). Over time, the stimulation paired with a training strengthens mechanisms of neural plasticity, enhancing functional outcomes through interaction with endogenous plasticity mechanisms (15), leading to greater and more lasting effects (16). When used in the context of aphasia, tDCS is directed to language regions and paired with SLT (17, 18). It has been widely used for language recovery after stroke (19) and in other conditions (20, 21). While it has been shown to be effective in stroke-induced aphasia (22), studies on its effects in PPA remain limited.

Initial studies in PPA have shown promise combining SLT with stimulation, using either transcranial magnetic stimulation (TMS) or tDCS (11, 23, 24). These studies have reported the treatment to be safe and well-tolerated, and to improve language outcomes compared to SLT alone (24–28). Interestingly, a recent meta-analysis reported that tDCS paired with behavioral therapies is more effective than TMS in improving naming ability (24) and it is easier to be integrated into clinical and research protocols for this application (24). This is likely attributed to the mechanism of tDCS, which delivers a mild electrical current, inducing sub-threshold alterations in the neuronal resting membrane potential (29), with its lasting effects associated with neuroplasticity processes (15, 30) and a broader engagement of the relevant networks (19, 31). However, despite the growing body of studies, a recent meta-analysis had insufficient data to conduct the planned network meta-analyses (17).

tDCS effects are cumulative (32, 33), and needs to be delivered in repeated daily sessions extending for weeks, or months to evaluate its potential for functional benefit. As a result, many studies have been limited in intervention dosing due to the requirement for patients to travel to a clinic to receive treatment (34). One advantage of tDCS is that the devices can be portable and wearable. This allows tDCS to be simultaneously paired together with SLT in real-time.

We have developed a home-based remotely supervised (RS)-tDCS protocol (35, 36), where tDCS is provided as a telehealth intervention using video visits. This increased access is particularly relevant to people living with conditions that make travel to appointments a burden, particularly people with PPA (37). Such interventions allow participants to receive treatment at home, from any U.S. location in sufficient dosing (38).

We evaluated our remotely supervised (RS)-tDCS protocol paired with SLT to help recover and preserve language function in individuals with PPA. By establishing a standardized home-based treatment protocol, we aimed to ensure a sufficient number of repeated sessions, facilitate rapid participant enrollment in clinical trials, and improve the efficiency of future clinical treatments.

In this study, we report the results of a 20-session intervention where tDCS was applied to the left inferior frontal gyrus (IFG) in combination with personalized word retrieval training through SLT. This was conducted in an open-label cohort of participants with PPA. While tDCS should be directed to the region that is activated during the training activity to enhance mechanisms of neural plasticity, the optimal brain target for different language therapies in PPA and variants is still inconsistent (24, 26, 39–42). Various brain targets have been employed in prior studies, namely the left dorsolateral prefrontal cortex (DLPFC) and the left frontotemporal region (11, 17, 24, 43). Given the distinct patterns of neurodegeneration in PPA, the IFG was selected as the stimulation target in this study because of its central role in language generation, encompassing lexical retrieval, syntactic processing, and speech production as these are the functions often primarily impaired in PPA, particularly in the nonfluent/agrammatic and logopenic variants. While the dorsolateral prefrontal cortex (DLPFC) is associated with executive functions and working memory and could be a valid target, its role is less directly linked to the core deficits in language production observed in PPA. The IFG's critical involvement in language processing and its proximity to regions of neurodegeneration in PPA make it a particularly compelling and clinically relevant target for intervention. Moreover, studies indicate that more favorable recovery occurs when patients predominantly activate regions where the neurodegeneration occurs, such as the left FG and left temporoparietal areas (43–45).

Our primary objective was to evaluate the feasibility of the home-based tDCS + SLT intervention. With many questions remaining, including the variant and severity of PPA most likely to benefit from the intervention, our goal is to have an established protocol that could be used for home-based, randomized sham-controlled trials to advance its optimization for clinical use.

## 2 Materials and methods

### 2.1 Study design and participants

The study was an observational, open-label case series studying an intervention of 20 daily home-based tDCS sessions paired with personalized word retrieval exercises performed via live video conference. All study procedures were approved by the Institutional Review Board Committee of New York University Grossman School of Medicine. The study was registered in November 2022 at clinicaltrials.gov (NCT05615922) and all participants provided electronic informed consent prior to their participation in any study procedure.

Eligibility criteria included 45–85 years of age (inclusive) and a confirmed diagnosis of PPA (logopenic or semantic variant, or unspecified) by the participant's treating neurologist. Participants with nonfluent/agrammatic PPA subtype or with another or additional language disorder were excluded, due to the specificity of the SLT protocol focus on naming.

To confirm premorbid cognitive functioning in at least the average range, and to ensure that participants had sufficient knowledge of the English language to understand and participate in the study procedures, we administered a test of single word vocabulary recognition, the Wide Range Achievement Test-Fourth Edition (WRAT-4) (46), Word Reading subtest. Participants were required to have a normative *z*-score −1.5. In the case that participants were not sufficiently fluent to complete the task of oral word reading, the Peabody Picture Vocabulary Test-4th Edition (PPVT-4) (47) was administered as an alternative nonverbal single word recognition test, employing the same cut-off score of ≥-1.5 SD for study entry. Potential participants were required to earn a *T* score of ≥20 in the Wechsler Adult Intelligence Scale-Fourth Edition (WAIS-IV) Matrix Reasoning subtest (48), serving as an index of current general cognitive functioning to exclude those with severe cognitive impairment. The WAIS-IV Matrix Reasoning subtest was utilized as a proxy for general cognitive functioning in this study, given its established role as a non-verbal measure of reasoning, problem-solving, and perceptual organization skills. This subtest has been employed in previous studies as a screening tool to exclude participants with severe cognitive impairment, typically using a *T*-score threshold of ≥20 (36, 38).

Additional exclusion criteria included history of an uncontrolled seizure disorder or a recent history of seizures (< 5 years), history of head trauma, had a head or neck medical device implanted, had skin disorders or sensitivity near the stimulation area, or were pregnant or breastfeeding. Stable internet access was also required for participation in the video visits.

Once enrolled, participants were shipped a study kit that included the tDCS device and were scheduled for a baseline visit. Prior to this baseline visit, participants identified a list of high frequency common nouns and verbs that they use in their daily life. This list of words was utilized to make a trained and untrained stimuli list. The remote baseline visit was then conducted for device orientation, training, and tDCS tolerability testing (see (34) for detailed procedures). Following the tDCS tolerability test, participants completed a language assessment and the study outcome measures. Initial findings were used to inform the daily treatment sessions.

The participants underwent the baseline cognitive and language assessment, including self-reported questionnaires about their language use and daily functioning and cognitive measures. All 20 daily intervention sessions were completed by the participant from home, and a research team member (either a speech-language pathologist or master's level neuropsychologist) connected in real time via videoconference (Zoom). At the end of the tDCS intervention, participants underwent the same assessment conducted at baseline.

### 2.2 Study equipment

**Figure 1:**
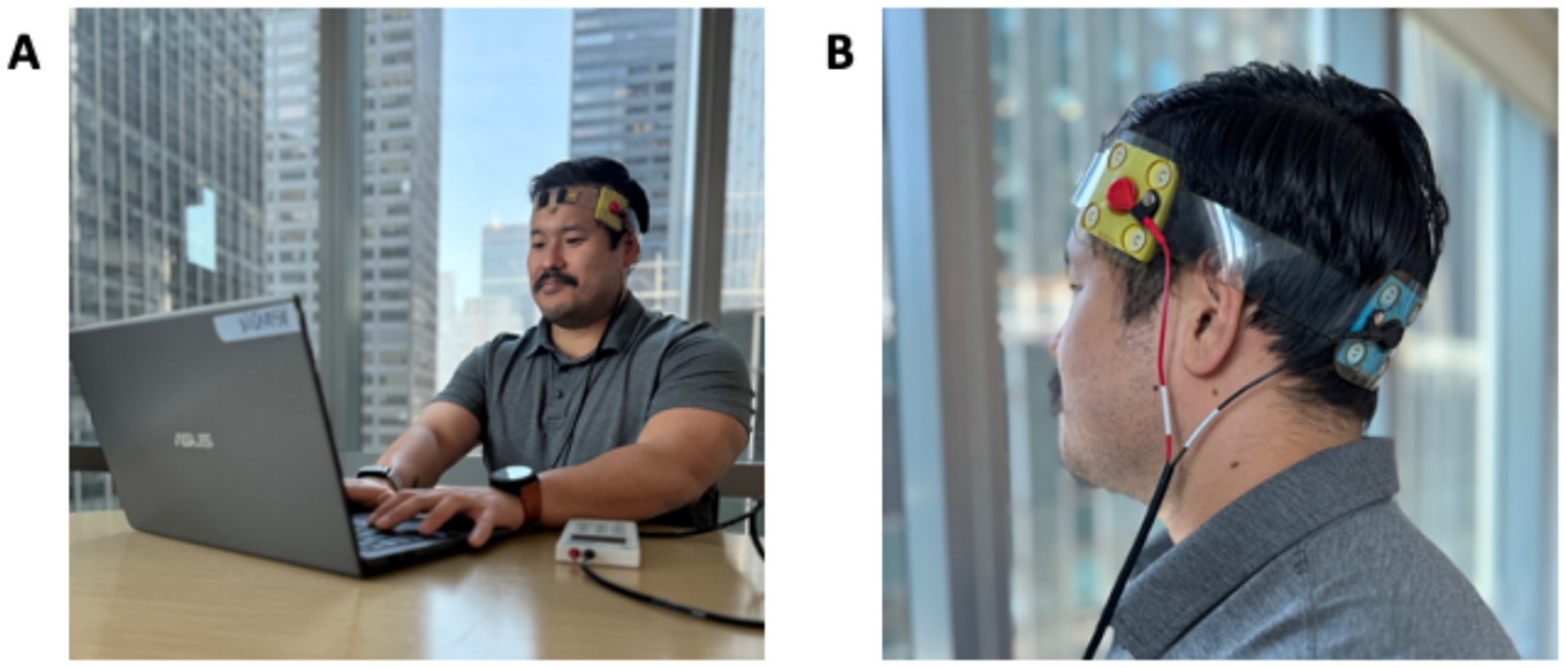
Example of the home-based RS-tDCS setup **(A)** and F7-O1 electrode montage **(B)**.

The 1 × 1 tDCS mini-clinical trial device (mini-CT; Soterix Medical Inc., Woodbury, NJ) was pre-programmed to deliver a constant direct electrical current at 2.0 mA for a duration of 30 min. Customized SNAPstrap headgear was used to target the left IFG, with the anode placed over F7 and the cathode over O1 (10–20 EEG system; Figure 1). Pre-saturated sponge electrodes (5 cm × 5 cm; SNAPpads) were utilized for easy and single use “snap” placement onto the headset during each session.

For this open-label pilot trial, the direct electrical current was ramped up to the target intensity of 2.0 mA over an initial 30 s, was delivered consistently at 2.0 mA for the following 29 min and was then ramped down to 0.0 mA over the final 30 s.

### 2.3 Intervention: tDCS and personalized word retrieval exercises

All participants completed 20 remotely supervised intervention sessions over 1 month (five sessions per week, Monday through Friday), pairing tDCS with personalized word retrieval exercises. The language training protocol followed a modified version of Lexical Retrieval Cascade Treatment (49). Prior to the baseline visit, participants, with caregiver support, selected 88 words and phrases of personal relevance (i.e., high familiarity and frequency) from a fixed list of 180 items. This list, differing for men and women, included everyday objects and actions across six semantic categories: clothes, hygiene, household items, places, food, and actions (50).

At baseline, participants viewed a PowerPoint presentation featuring images of their 88 selected target items and completed a picture naming task to assess spontaneous naming for each item. Based on their baseline performance, 30 words were chosen for training, and 30 served as untrained controls, with an equal distribution of items participants named incorrectly in both sets. The selection of target items prioritized words that were personally relevant yet challenging for participants to retrieve, excluding items already well-known to them. This personalized approach ensured that the intervention set reflected the participants' daily lives and language abilities, combining participant-reported high-frequency words with clinician-selected items to include a range of complexity.

The language training protocol followed a modified version of the Lexical Retrieval Cascade Treatment (49), as outlined by Henry et al. (49). Participants were presented with photos of target training items (common nouns and verbs) and prompted to verbally produce the names of the pictures. A word-naming cueing hierarchy was employed to facilitate word retrieval through self-cueing, beginning with semantic cues and progressing to orthographic and phonological prompts as needed. Regardless of whether participants correctly identified the stimulus, all cues within the hierarchy were presented to reinforce the representation of the target stimuli.

The word-naming approach was consistent across all participants and is detailed further in Table 1.

**Table 1:** Word naming practice cues.

Training step^*^	**Procedure**	**Instructions**
1. Semantic self-cue	Picture presented; patient prompted for semantic information and/or autobiographical information	What can you tell me about it? (Where do you find it? What is it used for? What is it made of?)
2. Orthographic self-cue	Prompt written form or first letter of word	Can you write the word? Can you write the first letter?
3. Phonemic self-cue	Prompt initial phoneme	What sound does that letter make? What is the first sound of the word?
4. Oral reading	If item not named, provide written form and participant reads aloud	Here is the word. Can you read it?
5. Written and spoken repetition	Participant writes and says the word three times	Now write and say the word (three times)
6. Yes/no question	Provide participants with yes/no questions	Examples: Is it sweet? Can you buy it at the supermarket?
7. Recall	Participant provides two semantic features and writes and says the word	Now tell me two important things about this item. What is this called? Can you write it?

^*^Adapted from Henry et al. (49).

### 2.4 Study outcomes

#### 2.4.1 Safely, tolerability and feasibility of the intervention

Feasibility was assessed using two primary measures: the capture and monitoring of adverse events (AEs) and the completion rate of the intervention. AEs were systematically recorded throughout all sessions for each participant, with any event necessitating session discontinuation or treatment termination categorized as treatment-limiting. Completion rate was defined as the successful completion of all scheduled intervention sessions.

#### 2.4.2 Language measures

We hypothesized, based on functional tDCS targeting, that participants would have greater improvement on the trained items. The preliminary efficacy of the intervention was measured by naming for trained vs. untrained naming items. During baseline testing, we evaluated spontaneous naming for each target and recorded the results. When creating the trained and untrained stimulus, each set of 30 items each, we aimed to balance the difficulty level by distributing an equal number of targets that were difficult for participants to retrieve between the two sets. For example, if a participant provided incorrect responses for 20 out of the 88 items, we allocated 10 of those targets to the untrained set and 10 to the training set. We then measured naming for each set at baseline and after the intervention.

#### 2.4.3 Additional language outcomes

Standard measures of aphasia were administered at baseline and repeated at the end of the intervention to explore generalized effects on language functions. These included the Quick Aphasia Battery (QAB) (50), the Boston Naming Test-Short Form (BNT-Short) (51), and the Controlled Oral Word Association Test (COWAT) (52). The QAB provides a reliable and multidimensional assessment of language function in a short period across eight language subtests; the QAB was adapted for remote use over Zoom. The BNT-Short form tests confrontational naming using 15 line-drawn pictures presented in increasing order of difficulty. The COWAT assesses verbal (phonemic and semantic) fluency. Participants are given 60 s to name words within a given category (phonemic and semantic).

#### 2.4.4 Self-reported outcomes

Additionally, self-report questionnaires assessing communication abilities and overall quality of life were administered at baseline and study end using REDCap (53). These included the Aphasia Communication Outcome Measure (ACOM) (54) and Stroke and Aphasia Quality of Life Scale-39 (55) (SAQOL-39), the Patient-Reported Outcome Measurement Information System (PROMIS) Ability to Participate in Social Roles and Activities (56), and the PROMIS Global—Physical Health and Mental Health categories (57).

#### 2.4.5 Caregiver perspectives

For the participants that included caregiver support during the intervention procedures, we provided caregivers with the opportunity to provide their perspectives through open-ended prompts at study end.

### 2.5 Statistical analysis

Data were analyzed using SPSS version 28.0 (IBM Corp, Armonk, NY). Descriptive statistics (mean ± standard deviation) were calculated to determine participants' demographic and clinical characteristics. The Kolmogorov–Smirnov test was used to assess data normality. The value obtained for QAB, COWAT phonemic and semantic conditions, BNT-Short, and the numbers of correct trained and untrained words were compared using paired sample *t*-test for differences between baseline and end of intervention. Type I error (α) was set at 0.05.

To assess the adequacy of the sample size for detecting changes in the primary outcome, a retrospective power analysis was conducted. Based on a mean improvement of 8.0 in trained naming items, with a pooled standard deviation of 7.9 and an alpha level of 0.05, the observed effect size (Cohen's *d*) was 1.00. Using a sample size of 10 participants, the achieved power was 80.5%. While this suggests sufficient power to detect within-group changes, the absence of a sham-controlled condition limits the interpretation of these results, emphasizing the need for larger and more rigorously designed trials in future studies.

All analyses were conducted on an intention-to-treat basis. For participants with missing outcome data at the intervention end, the last observation carried forward (LOCF) method was used to impute missing values. This approach was chosen to maintain consistency in the dataset while minimizing bias in the analysis of this feasibility study. As no major attrition occurred and all participants completed the intervention, the use of imputation was minimal. Future studies with larger sample sizes may consider multiple imputation methods to more robustly address missing data.

## 3 Results

**Figure 2:**
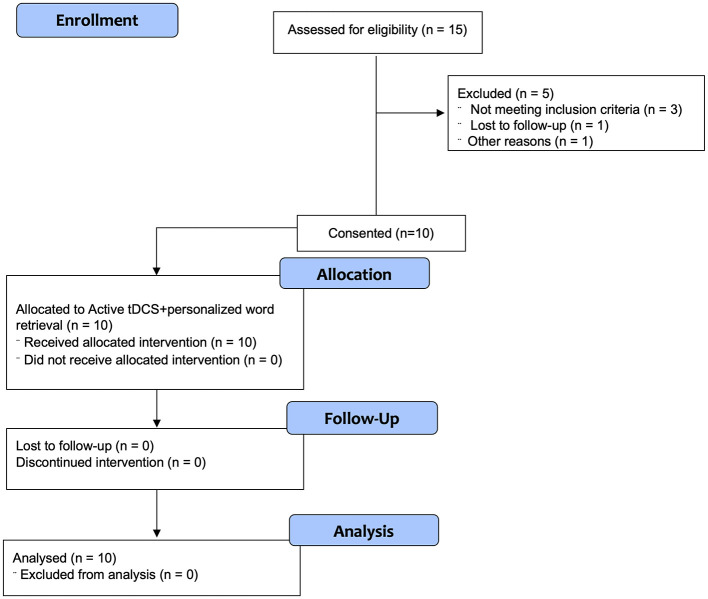
Consort diagram for open-label trial.

Participants were nationally recruited through clinicaltrials.gov and were sequentially enrolled to reach the target of ten participants. A total of *n* = 15 participants were screened, with *n* = 3 failing to meet the inclusion/exclusion criteria [specifically, they did not pass the Peabody Picture Vocabulary Test (PPVT)], *n* = 1 participant was lost to follow-up, and *n* = 1 participant was screened after the target enrollment was reached. This resulted in *n* = 10 participants consented and enrolled in the study (Figure 2), four women and six men, ages 56–82 years (M age = 70 ± 6.90 years). All participants were native English speakers, including one who was bilingual in English and French and represented seven different U.S. states in their home location. All participants had neurologically confirmed diagnosis of PPA, with either logopenic variant (lvPPA; *n* = 2), semantic variant (svPPA; *n* = 2), or unspecified (*n* = 6, PPA) and with prominent conversational word-finding problems. All ten participants identified their race as “White, not of Hispanic Origin”; no participants identified their ethnicity as “Hispanic or Latino.”

At screening, n = 5 participants met eligibility criteria using the WRAT-4 Word Reading subtest (mean z-score = −0.04 ± 0.90) and n = 5 used the PPVT-4 (mean z-score = −0.81 ± 0.29). All n = 10 participants met eligibility criteria on the WAIS-IV Matrix Reasoning subtest, scoring in the in the average range or higher (mean T score 45.70 ± 5.27, range 40–53).

### 3.1 Feasibility of the intervention

In this fully remote trial, all 10 participants were able to complete the home-based procedures, with 7/10 (70%) participants requiring caregiver assistance to complete the study procedures and daily intervention sessions. Consistent with the broader literature supporting the safety of tDCS (36), there were no serious adverse events across the study. As would be expected, participants reported mild sensations of tingling and warmth at the initiation of the sessions. No session for any participant was discontinued due to tolerability (e.g., per protocol, discomfort rating of 7/10 or higher), showing that the procedures were well-tolerated.

All participants successfully completed all scheduled intervention sessions, resulting in a 100% completion rate and no attrition. Consistent with prior studies, there were no serious adverse events (AEs) reported during the intervention. Mild, self-limited sensations such as tingling at the electrode site were reported by two participants but did not disrupt adherence or require further intervention. These outcomes demonstrate that the intervention is both practical and well-tolerated in a home-based setting.

### 3.2 Language outcomes

**Figure 3:**
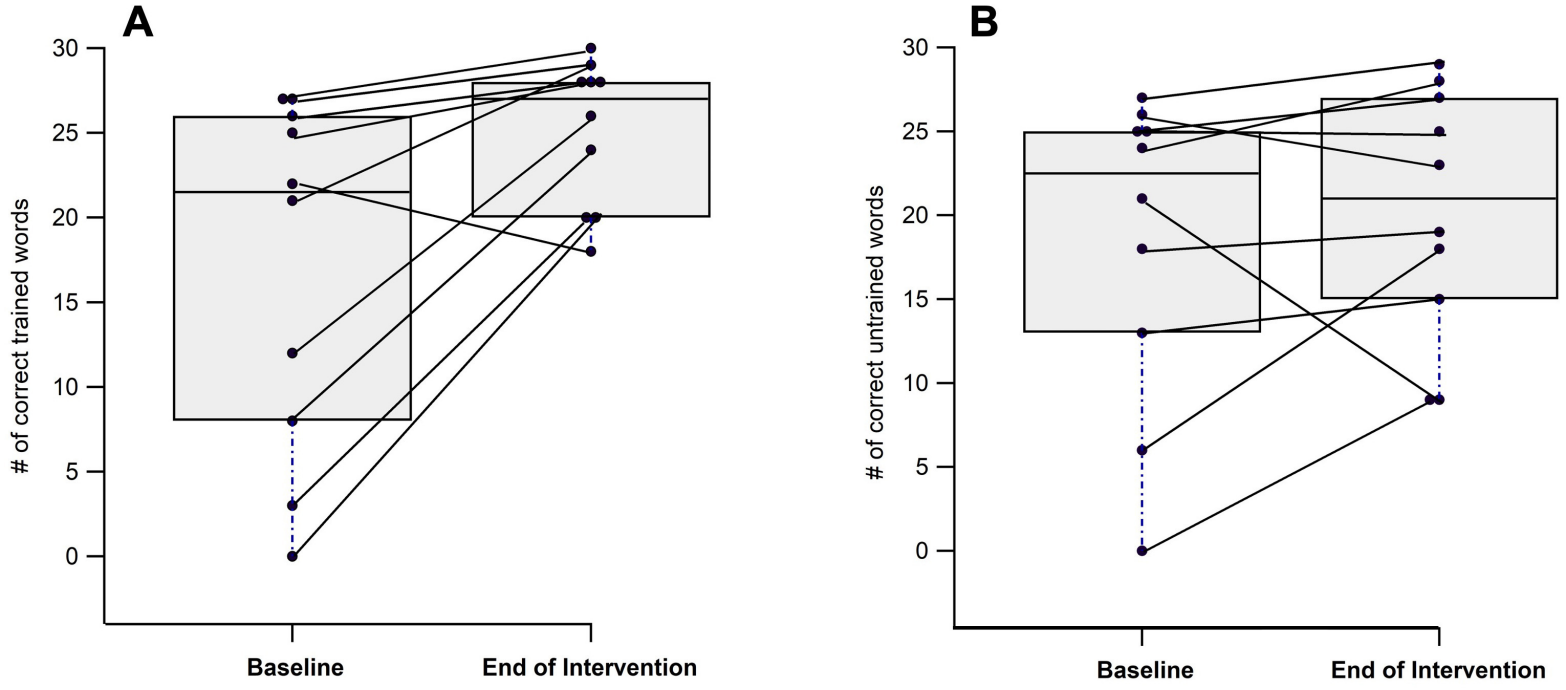
Individual and group differences from baseline to end of intervention in naming trained **(A)** and untrained **(B)** words.

At the end of the intervention, participants correctly named significantly more trained items compared to baseline (17.10 ± 10.42 vs. 25.10 ± 4.33, p = 0.014) (Figure 3A), while no significant change was found for the untrained items (18.50 ± 9.30 vs. 20.20 ± 7.45; Figure 3B; Table 1).

### 3.3 Additional language outcomes

The participants significantly improved in naming on the BNT-Short form (6.20 ± 5.55 vs. 7.80 ± 5.18, p = 0.016). Other language outcomes were stable with no significant decline in language functioning across time points (Table 2).

**Table 2:** Change from baseline to end of intervention on language measures.

	**Baseline**		**Intervention end**		***df***	***t***	***p***	**Cohen's *d***
	**Mean**	**SD**	**Mean**	**SD**				
QAB total	9.00	1.70	9.14	1.39	9	−0.65	0.531	0.21
COWAT (F-A-S)	20.90	17.28	22.30	14.97	9	−0.76	0.469	0.24
COWAT (animals)	6.30	4.00	7.60	5.54	9	−1.18	0.270	0.37
BNT-short form	6.20	5.55	7.80	5.18	9	−2.95	0.016^*^	0.93
Trained items	17.10	10.42	25.10	4.33	9	−3.05	0.014^*^	0.96
Untrained items	18.50	9.30	20.20	7.45	9	−0.83	0.428	0.26

COWAT, Controlled Oral Word Association Test (51); QAB, quick aphasia battery (49); BNT-Short, Boston Naming Test Short Form (50).

^*^Indicates statistically significant difference p < 0.05.

### 3.4 Self-reported outcomes

In addition to language measures, participants completed self-report assessments at baseline and at the end of the intervention to evaluate their experience and perceived benefits of the intervention. As shown in Table 3, while participants reported subjective trends toward improvement in their communication abilities (ACOM-26), social participation (PROMIS), and quality of life related to their aphasia (SaQOL), none of these changes reached statistical significance. Notably, participants reported a global health benefit (PROMIS), with a moderate effect size observed for aspects of mental health and wellbeing. These findings suggest potential perceived benefits of the intervention; however, the lack of statistical significance highlights the need for cautious interpretation of these trends.

**Table 3:** Comparison of baseline and post-intervention outcomes across self-report measures.

	**Baseline**		**Intervention end**		***df***	***t***	***p***	**Cohen's *d***
	**Mean**	**SD**	**Mean**	**SD**				
ACOM-26	56.05	7.44	56.79	6.67	9	−0.295	0.775	0.10
PROMIS social roles	53.30	9.13	52.10	5.29	9	0.332	0.748	0.11
SaQOL	4.14	0.32	4.07	0.52	9	0.479	0.644	0.16
PROMIS global—physical health	41.60	4.74	42.47	5.63	9	−0.461	0.656	0.17
PROMIS global—mental health	47.59	2.45	45.92	4.03	9	1.233	0.249	0.39

ACOM, Aphasia Communication Outcome Measure (54); PROMIS Global Health, Patient-Reported Outcomes Measurement Information System (PROMIS)–Global Health (57); PROMIS Social Roles and Activities, Patient-Reported Outcomes Measurement Information System Social Roles and Activities (56); SaQOL, Stroke and Aphasia Quality of Life-39 Item (55).

### 3.5 Caregiver perspectives

For those participants relying on caregivers for study procedures (n = 7), their caregivers also provided perspectives concerning the response to intervention. Overall, there was a strong belief in the positive effects of the intervention on the participants. They reported that the intervention was beneficial and even life-changing for the participants, with some pursuing continued tDCS treatment for their participant. Caregivers observed that the participants either remained stable or showed slight improvement during the intervention. They also noted that since the study ended, the participants have been struggling more with daily tasks and communication. The caregivers are seeking advice on how to proceed with the participants' care, especially as the participants who encounter difficulty leaving their homes or traveling to receive treatment.

## 4 Discussion

In this study, our primary objective was to investigate the feasibility of a one-month home-based intervention using our RS-tDCS protocol, combining 20 daily sessions of tDCS with personalized word retrieval training in individuals living with PPA. Our approach was informed by the safety and tolerability of tDCS (58), which has been well-established in previous research and validated for home use through our telehealth remotely supervised protocol (36).

All participants completed the protocol, demonstrating the feasibility of the home-based intervention for individuals with PPA. The intervention was well-tolerated across all participants, with no reports of treatment-limiting adverse events. This feasibility meets an important objective in demonstrating that participants with PPA can be reached at home to receive a standardized intervention of tDCS paired with SLT. With the high completion rate of all 20 intervention sessions across participants, we can use this protocol for the critical next steps of controlled trials (17, 59).

Following the 1-month intervention, all participants performed better in naming trained vs. untrained items, with a significant group improvement in confrontation naming from baseline to the end of the intervention. These findings suggest that the combined approach of tDCS and personalized word retrieval training may be effective in offsetting lexical retrieval decline in PPA (60, 61). All language functions measured either were found to be improved or maintained.

At the intervention end, the participants reported subjective improvements in communication associated with increased ability to participate socially and corresponding quality of life related to their aphasia. They also reported global increases in health, particularly in mental health, reflecting the meaningfulness of even minor benefits of an intervention for people with PPA. Caregivers provided qualitative feedback that underscored the accessibility of the intervention and highlighted noticeable improvements in participants' communication abilities. They also reported secondary benefits to the participants' overall wellbeing, further supporting the perceived meaningfulness of the intervention in daily life.

The promising results of this study are consistent with what has been reported to date across trials of PPA with tDCS (17). Given the absence of treatment options for people currently diagnosed with PPA and the home-based access to our treatment, we met unprecedented demand for our trial. As reflected by the patient participants' caregivers in our trial, there is an urgent need to rapidly move forward to evaluating this treatment approach.

In addition to its practical and preliminary therapeutic potential, our approach highlights the importance of incorporating sequential assessments and individual lexical context in the design of interventions for populations with diseases lacking curative treatments, such as PPA. Personalized and contextually relevant lexical training not only maximizes the use of preserved language abilities but also fosters a sense of agency and meaningful progress for individuals. Sequential assessments, although necessary for evaluating intervention outcomes, may also serve as a motivational tool, reinforcing participants' engagement in the treatment process. These considerations underline the importance of tailoring interventions to the unique needs of individuals, particularly in the absence of curative options.

The feasibility of our RS-tDCS protocol supports the next step of conducting home-based sham-controlled trials to rigorously evaluate the specific contributions of tDCS to the observed outcomes. Future controlled trials are essential to confirm the benefits of tDCS when paired with SLT, while also refining the intervention through comparisons of different montages and dosing regimens (35, 59). For example, targeting specific brain regions in accordance with distinct PPA subvariants may yield greater therapeutic outcomes (39). Larger-scale trials will also enable identification of baseline clinical characteristics that predict treatment success, allowing for a more personalized and effective approach to paired tDCS and SLT interventions.

Additionally, the home-based nature of the protocol facilitates high throughput, with rapid enrollment and completion of intensive intervention periods, further supporting its potential for clinical implementation (35, 59). Recent studies, such as Neophytou et al. (62), have further demonstrated the feasibility, safety, and acceptability of home-based tDCS protocols in patients with PPA, reinforcing the potential for scalable and accessible treatments in this population. These findings align with the broader application of home-based tDCS in other neurological conditions, including stroke, Parkinson's disease, and multiple sclerosis, where studies have shown its practicality and preliminary efficacy (35, 59). Expanding this evidence, our findings add to the growing support for remote interventions that can overcome barriers to in-clinic treatments, particularly for individuals with chronic neurodegenerative conditions who face mobility and logistical challenges.

Future research should aim to establish standardized protocols for home-based tDCS delivery across diverse neurological conditions, enabling comparisons across patient populations to identify shared benefits and address the unique needs of individuals with PPA. This initial study had several limitations, including its open-label design and the absence of a sham treatment arm, which limits the ability to isolate the specific effects of tDCS from SLT or non-specific factors like placebo or practice effects. While this study was designed to assess feasibility rather than efficacy, these findings are preliminary. Future sham-controlled trials are essential to rigorously evaluate the efficacy of tDCS in improving naming performance and to determine its added value when combined with SLT. Additionally, the lack of blinding for outcome assessments increases the risk of assessor bias, which could have influenced the observed results. Future trials should incorporate blinded assessors to enhance the validity and reliability of findings.

We also included a relatively small cohort, with different clinical characteristics including patients with both logopenic and semantic variants. While this cohort informs and confirms the feasibility of our intervention, as well as providing initial evidence for its effectiveness, larger studies with more diverse participants are required to validate the generalizability of our findings. In particular, the small sample size limits our ability to detect more subtle effects or assess outcomes across a broader range of measures, further emphasizing the need for larger-scale studies with robust recruitment strategies.

Another potential limitation is the dosing parameters for the tDCS intervention may not have been the most optimal for the intervention. We selected the parameters for the intervention, including the IFG montage and 30 min at 2.0 mA intensity, based on standards and evidence to date (13, 29, 59, 62). However, individual variations in response to tDCS and the ideal dosing parameters may exist, potentially affecting treatment outcomes. Further investigation into various dosing protocols and personalized approaches to administering tDCS is necessary to enhance its effectiveness in individuals with PPA. Additionally, while we utilized the WAIS-IV Matrix Reasoning subtest as a proxy for general cognitive functioning, we acknowledge its limitations as a comprehensive indicator of cognition. This subtest primarily evaluates non-verbal reasoning, perceptual organization, and problem-solving skills, and does not assess other important cognitive domains such as memory, attention, or verbal fluency. Despite its limited scope, the use of the WAIS-IV Matrix Reasoning subtest is consistent with previous studies employing a *T*-score threshold of ≥20 to exclude participants with severe cognitive impairment. Future studies should incorporate a broader battery of cognitive tests to provide a more comprehensive assessment of general cognitive abilities in this population.

The inclusion of neuroimaging data could also include further insight into the specificity of treatment targeting and clinical responses as well as advancing our overall understanding of its mechanisms of benefit. Finally, the short-term nature of the intervention and the follow-up period in our study restrict our ability to evaluate the long-term effects of the combined tDCS and word retrieval training. Future research with extended follow-up periods is essential to assess the sustainability of treatment effects and the potential preservation of language function over time. Additional studies are needed to evaluate the independent contributions of tDCS and the speech-language intervention to treatment gains.

Despite its limitations, this study highlights the feasibility and potential effectiveness of a home-based tDCS intervention paired with personalized word retrieval training for individuals with PPA. Addressing these limitations in future research will be critical for validating and optimizing this combined approach.

## 5 Conclusions

This one-month, home-based intervention combining tDCS with personalized naming training was accessible and feasible for individuals with PPA. This cohort has successfully validated a protocol for delivering tDCS paired with individualized language training intervention at home for individuals with PPA. The findings of language improvement and positive clinical outcomes in our cohort urgently warrant further investigation of this promising intervention.

## Data Availability

The raw data supporting the conclusions of this article will be made available by the authors, without undue reservation.
